# Timely Initiation of Breast Feeding and Associated Factors among Caesarian Section Delivered Mothers in Health Facilities of Dessie City Administration, North Eastern Ethiopia

**DOI:** 10.3390/pediatric13010001

**Published:** 2020-12-23

**Authors:** Roza Shiferaw, Sisay Eshete Tadesse, Tefera Chane Mekonnen, Aregash Abebayehu Zerga

**Affiliations:** 1 Dessie Zonal Health Department, Dessie 6000, Ethiopia; rozashiferaw2006@gmail.com; 2 School of Public Health, College of Medicine and Health Sciences, Wollo University, Dessie 1145, Ethiopia; teferachane@gmail.com (T.C.M.); aregusina@gmail.com (A.A.Z.)

**Keywords:** timely initiation breastfeeding, early initiation breastfeeding, breast feeding, cesarean section, factors, cross-sectional, Dessie

## Abstract

*Objective*: To assess the magnitude and associated factors of timely initiation of breastfeeding among cesarean section delivered mothers. *Methods*: A health facility-based cross-sectional study was employed among 421 systematically selected mothers from February to June, 2017. Data were collected by a structured questionnaire. Data entry and analysis was done using Epi Data and SPSS version 24. Binary logistic regression was computed to identify factors. Adjusted odds ratio with a 95% confidence interval was used to declare statistical significance. *Result*: The magnitude of timely initiation of breast feeding (among mothers who gave birth by cesarean section was 57%. Counseling during antenatal care (AOR = 3.32; 95% CI: 1.80, 6.13), facility where cesarean section (CS) was performed (AOR = 2.55; 95% CI: 1.57, 4.14), and post-CS counseling (AOR = 6.93; 95% CI: 3.99, 12.02) were factors that contributed for the practice of timely initiation among cesarean section delivered mothers. *Conclusions*: The magnitude of TIBF was good. Counseling during ANC, the facility where CS was performed and post-natal advice were factors associated with TIBF. Implementation of baby-friendly hospital initiatives should be strengthened in order to promote timely initiation of breast feeding.

## 1. Introduction

According to the World Health Organization (WHO) and the United Nations Children’s Fund (UNICEF) recommendation, mothers should initiate breast feeding within one hour of birth [1,2]. After birth, the suckling reflex of a baby becomes the most active and more alert during the first 30–60 min [3]. This will stimulate the production of breast milk and ensures more intake of colostrum, highly nutritious food, produced during the first few days after birth [4,5,6]. Timely initiation of breast feeding has benefit for both mother and her child. For the child, it creates a special bond with the mother, protects him against common childhood illnesses, enhances dental and brain development and has long-term health benefits, such as reducing the risk of becoming overweight and obese in childhood and adolescence [7,8,9]. For the mother, it facilitates the return of the uterus to its pre-pregnancy size, reduces risk of breast, ovarian and uterine cancers, decreases the risk for osteoporosis and enhances emotional health [4].Despite the lifelong health benefits, only 39% of mothers of newborns in developing countries initiate breast feeding within an hour of birth. The majority of this is contributed by Africa (47%) and Asia (31%) [10]. Only 58% of mothers in Ethiopia initiate breast feeding within an hour of giving birth [11].

Globally, over 1 million neonates die each year on the day they are born [10,11,12]. Among the various reasons, neonatal hypothermia was the major contributor, which is mainly happening due to the delayed initiation of breastfeeding and infection. Timely initiation of breastfeeding protects the newborn from acquiring infections and reduces newborn mortality [10]. Timely initiation of breast feeding can prevent 22% of neonatal deaths [13].

Cesarean section (CS) delivery was one of the factors that delayed breastfeeding initiation and decreased its continuation [12,14]. Currently, the rate of CS delivery is rapidly increasing in many low- and middle-income countries [15,16].

Despite the emphasis given and efforts made by the government and other stakeholders, timely initiation of breast feeding was low in Ethiopia [11]. The neonatal mortality in Ethiopia was 30/1000 [17]. The prevalence of timely initiation of breast feeding (TIBF) among CS delivered mothers in Ethiopia reaches 47.4% [5,18]. Mode of delivery, particularly CS delivery, is widely believed to adversely affect breast feeding, but individual population studies examining the prevalence and associated factors between cesarean delivery and breast feeding are inconsistent [19]. Regardless of research efforts, there is still limited information on timely initiation of breast feeding among CS delivered mothers. Assessing the prevalence of early initiation of breast feeding among CS delivered mothers is relevant for taking intervention measures and promoting it. Therefore; the aim of this study was to assess the magnitude of timely initiation of breast feeding and its associated factors among CS delivered mothers at health facilities of Dessie city administration.

## 2. Methods and Materials

### 2.1. Study Area, Design and Period

A health facility-based cross-sectional study design was employed among mothers who gave birth by CS in health facilities of Dessie city administration. The study was conducted from 1 February–30 June 2017. Dessie town is located 401km to the north east of Addis Ababa and 480 KM east of Bahir Dar the capital of Amhara Regional State. The administrative city has a total population of 216,384. The sex composition of the town is 101,400 (47%) male and 114,984 (53%) female. There are 33 health facilities that provide curative focused health service. Of these, only six health facilities are providing cesarean section. Based on the data obtained from the city administrative health office department, on average there were 1542 mothers who gave birth by cesarean section in the 2015/16 year.

### 2.2. Inclusion and Exclusion Criteria

Mothers who gave birth by CS with live birth in health facilities of Dessie city administration during the study period were included in the study, while neonate with gross congenital anomalies (anomalies that can cause failure to suck breast milk) and mothers with critical illnesses were excluded from the study.

### 2.3. Sample Size Determination and Sampling Procedure

The sample size was calculated using single population proportion formula. The assumptions for calculating the sample size were: proportion of mothers with timely initiation of breast feeding among CS deliveries, 47% (10), a confidence level of 95% and a margin of error of 5%. So, the sample size was calculated as *n* = (1.96)^2^ × 0.47 × (1 − 0.47)/(0.05)^2^ = 382.8 = 383. By adding a 10% non-response rate, the total sample size was 421.

All health facilities that provide CS service in Dessie city administration were included in the study. The sample size was proportionally allocated to each health facility. All mothers who gave birth by CS with live infants were counted by a systematic sampling technique, i.e., every two mothers with their neonate.

### 2.4. Operational Definition

Timely initiation of breast feeding: is the initiation of breast feeding within an hour after birth for mothers who gave birth by CS under spinal anesthesia. For mothers who have had a cesarean section under general anesthesia, timely initiation of breast feeding is considered if mothers initiate their neonate breast feeding as soon as they recover from general anesthesia [20,21].

### 2.5. Data Collection Tool and Quality Control

Data were collected using a structured interviewer administered questionnaire. The questionnaire was adapted from previous relevant literature [12,22,23]. The questionnaire was first translated to Amharic and then back to English. It consists of three parts (socio-demographic data, cultural and economic factors and obstetrical and medical factors). Pre-testing was performed on 5% of mothers who delivered by CS at Wodia Hospital before the actual data collection by six data collectors with BSc degrees (four midwifery nurses and two health officers were recruited). The interview was carried out one hour after delivery if the mother was awake from the anesthesia or before her discharge from the facility.

Data collectors and supervisors were trained by the principal investigator for two days on their duties and responsibilities (confidentiality, consent form and on how to interview the study participants), objectives of the study and clarity of questions. Supervisors were responsible for supervising and coordinating data collectors and communicate with the principal investigator. The overall process of data collection was monitored by the principal investigator.

### 2.6. Data Processing and Analysis

Data were coded and entered using Epi Data version 3.1 and transferred to SPSS version 23 for analysis. Descriptive statistics was computed and the result was described by mean (standard deviation), frequencies and percentages. Bi-variable binary logistic regression was conducted and variables having a *p*-value < 0.25 were selected for multivariable logistic regression. Adjusted odds ratio with 95% confidence interval and a *p*-value < 0.05 were used to declare the statistically significant association between TIBF and the independent variables. Multi-collinearity was checked using standard error and the model’s goodness of fit was checked using Hosmer-Lemeshow test.

### 2.7. Ethical Consideration

Ethical clearance was obtained from the Institutional Review Board (IRB) of College of Medicine and Health science, Wollo University. After ethical clearance, a letter of support was obtained from Dessie city administration’s health department. The letter of support was submitted to six health facilities and discussions were held with the officials of the respective health facilities. Confidentiality was maintained during the data collection process. Written consent was obtained from each study subject before interviewing them. Those mothers who did not initiate breast feeding were given nutrition education.

## 3. Result

### 3.1. Socio-Demographic Characteristics

A total of 421 CS delivered mother–infant pairs were involved in the study, making a response rate 100%. Three hundred thirteen (74.3%) of the respondents were urban residents. More than half 219 (52%) of the neonates’ sex was male. With respect to their educational status, 34 (8.1%) were unable to read and write and 119 (28.3%) were in high school (Table 1).

### 3.2. Obstetrics & Medical Factors

All 421 (100%) respondents attended ANC during their pregnancy. two hundred seventy one (64.4%) of them got counseling during the ANC follow up. Of the total respondents, 281 (66.7%) have got advice on TIBF during their ANC visit. Of the total respondents, 312 (74.1%) experienced maternal chest-neonatal body contact within an hour of CS delivery (Table 2).

### 3.3. Information about TIBF and Cultural Factors

More than three fourths (339 (80.5%)) of the respondents had knowledge about timely initiation of breast feeding. More than half (240 (57%)) of the study participants initiated breast feeding to their neonate within one hour of CS delivery. The majority (390 (92.6%)) of mothers realized that colostrum was important for their neonate, of which 347 (82.4%) provided colostrum to their new born baby (Table 3).

### 3.4. Factor Associated with Timely Initiation of Breast Feeding

In the multivariable binary logistic regression analysis counseling during ANC follow up (AOR = 3.32; 95% CI: 1.80, 6.13), facility where cesarean section performed (AOR = 2.55; 95% CI: 1.57, 4.14), and post-cesarean section counseling were factors associated with timely initiation of breast feeding (AOR = 6.93; 95% CI: 3.99, 12.02) (Table 4).

## 4. Discussion

The Ethiopian infant and young child feeding guidelines include timely initiation of breast feeding as one of the recommendations for newborn care. The aim of this study was to assess the magnitude and associated factors of timely initiation of breast feeding among mothers who gave birth by cesarean section in health facilities of Dessie city administration. The study revealed that the magnitude of timely initiation of breast feeding among mothers who gave birth by cesarean section was 57%. This finding was similar to the report of Ethiopian demographic and health survey 2011 and a study conducted in Debre Birhan [11,12]. However, it was higher than studies conducted in Addis Ababa, Saudi Arabia [5,14,24,25,26] and India [27], while it was lower than a study conducted in Tigray [28]. This variation might be due to the implementation of baby-friendly hospital initiatives and mode of delivery [21].

Counseling during antenatal care follow-up was found to be statistically significantly associated with timely initiation of breast feeding. Those mothers who received counseling on TIBF as part of the antenatal care service were three times more likely to initiate breast feeding on time, as compared to those mothers who did not get counseling during their ANC service follow-up. This finding was consistent with a study done in India, which stated that counseling during ANC had a positive effect on timely initiation of breast feeding [26,27]. This might be because the information given during the antenatal care follow-up equips mothers with knowledge and skills that help them to start breast feeding within the first hour of giving birth. Meanwhile, a study from Goba district indicated that counseling had no effect on timely initiation of breast feeding [29]. This difference might be because of knowledge difference and the approach of health care professionals who gave counseling for pregnant women.

The place of delivery was also a significant factor for timely initiation of breast feeding among mothers who gave birth by cesarean section. Mothers who gave birth by CS at public health facilities were almost three times more likely to initiate breast feeding than those who gave birth at private health facilities. This finding was similar with a study conducted in Shiraz-Iran, which revealed that those mothers who gave birth at public health facilities initiated breast feeding in the first hour of birth [30]. This might be because of the implementation of baby-friendly hospital initiatives in public health facilities [31].

Post-CS advice for mothers by a health care provider on timely initiation of breast feeding within an hour of CS delivery is a significant factor in predicting timely initiation of breast feeding [32]. Mothers who have received advice post-CS were almost seven times more likely to initiate breast feeding on time, as compared to their counterparts. This finding is consistent with a previous study conducted in Goba, southeast Ethiopia [25,33]. This could be because mothers received information and support after the operation, helped them to have skin to skin contact. This will facilitate the initiation of breast feeding as soon as they gave birth.

In conclusion, the magnitude of early initiation of breast feeding was low. Counseling received during the ANC follow-up, the facility where CS was performed and the advice about TIBF within an hour of CS delivery by health care providers were factors associated with timely initiation of breast feeding among CS delivered mothers. Therefore, baby-friendly health facility initiatives need to be strengthened and implemented in both public and private health facilities. Health care providers working in both public and private health facilities need to be trained on the benefits, practice and support of timely initiation of breast feeding, especially in private health facilities.

## Figures and Tables

**Table 1:** Socio-demographic characteristics of mothers who delivered by CS, Dessie city administration, June 2017.

Variables		Frequency	Percent
Age of mother	18–24 years	96	22.8
	25–29 Years	211	50.1
	30–49 Years	114	27.1
Sex of neonate	Male	219	52.0
	Female	202	48.0
Residence	Urban	313	74.3
	Rural	108	25.7
Religion	Christian	180	47.8
	Muslim	241	52.2
Maternal education	Unable read & write	34	8.1
	Read and write	41	9.7
	Elementary	117	27.8
	High School	119	28.3
	Diploma and above	110	26.1
Maternal occupation	House wife	269	63.9
	Trader	59	14.0
	Civil servant	93	

**Table 2:** Obstetrics and medical characteristics of mothers who delivered bycesarean section(CS), Dessie administrative town, June 2017.

Variables		Frequency	Percent
Counseling during ANC follow up	Yes	150	35.6
	No	271	64.4
Frequency of ANC visit	≤2 times	8	1.9
	Three times	80	19
	≥above	333	79.1
TIBF information	Yes	140	33.3
	No	281	66.7
TIBF advise	Yes	286	67.9
	No	135	32.1
Conscious during CS delivery	Yes	260	61.8
	No	161	38.9
Time of chest contact with your neonate	Within one hour	312	74.1
	After one hour	109	25.9
Health facility where CS was performed	Governmental	241	57.2
	Private	180	42.8
Known maternal health problem	Yes	59	14
	No	362	86

**Table 3:** Awareness about timely initiation of breast feeding (TIBF) and cultural characteristics of mothers who delivered by CS, Dessie administrative town, June 2017.

Variables		Frequency	Percent
Knowledge about breast feeding initiation time	Within one hours	339	80.5
	>an hour of birth	82	19.5
Timely initiation of breast feeding	Yes	240	57.0
	No	181	43.0
Reasons for not practicing TIBF	Awareness problem	15	3.6
	Fear of operation wound	128	30.4
	Mother has no enough milk	34	8.1
	Maternal or neonatal problem	4	1.0
Colostrum importance to the neonate	Yes	390	92.6
	No	31	7.4
Colostrum given to the neonate	Yes	347	82.4
	No	74	17.6
TIBF importance to the neonate	Yes	407	96.7
	No	14	3.7
Source of information about TIBF	From health professionals	293	69
	From friend or workmate	104	24.7
	From public media	10	2.4

**Table 4:** Factors affecting timely initiation of breast feeding among CS delivered neonate pairs, Dessie city administration, June 2017.

Variable		EIBF		COR (95% CI)	AOR (95% CI)
		Yes	No		
Maternal Education	Not read and write	24(70.6%)	10 (29.4%)	0.34 (0.15, 0.79)	
	Read and write	34(82.9%)	7(17.1%)	0.17(0.07, 0.42)	
	Elementary	79(67.5%)	38(32.5%)	0.40 (0.23, 0.69)	
	High school	53(44.5%)	66(55.5%)	1.04 (0.62, 1.75)	
	College and above	50(45.5%)	60(54.5%)	1	
Husband Education	Not read and write	13(5.4%)	8(4.4%)	0.48 (0.19, 1.23)	
	Read and write	29(12.0%)	10(5.5%)	0.27 (0.12, 0.59)	
	Elementary	66(27.5%)	27(14.9%)	0.32 (0.19, 0.56)	
	High school	63(26.3%)	48(26.5%)	0.59 (0.37, 0.98)	
	College and above	69(28.8%)	88(48.6%)	1	
Residence	Urban	163 (51.2%)	150 (47.9%)	2.29 (1.43, 3.67)	
	Rural	77(71.3%)	31(28.7%)	1	
Counseling during ANC follow up	Yes	219(91.3%)	107(59.1%)	7.12 (4.16, 12.18)	3.32 (1.80, 6.13)
	No	21(7.7%)	74(40.9%)	1	1
Type of health facility where CS is performed	Gov’tal	186(77.5%)	85(46.9%)	3.94 (2.58, 5.99)	2.55 (1.57, 4.14)
	Private	54(22.5%)	96(53.1%)	1	1
Post CS counseling	Yes	215(89.6%)	81(44.8%)	10.62 (6.39, 17. 63)	6.93 (3.99, 12.02)
	No	25(10.4%)	100(55.2%)	1	1
Parity	<2	166(69.2%)	142(74.5%)	1.63 (1.04, 2.54)	
	>2	74(30.8%)	39(25.5%)	1	

Key: *p* < 0.05 with 95% CI is the level of significance.

## Data Availability

The data is available from the corresponding author on reasonable request.
